# Immunomodulatory effects of *Triatoma dimidiata* feces on *Trypanosoma cruzi* infection in a murine model

**DOI:** 10.1590/S1678-9946202567005

**Published:** 2025-02-03

**Authors:** Sergio Escobar-Laines, Victor Monteon, Carlos Ramírez-Sarmiento, Verónica Macedo-Reyes, Floribeth León Pérez

**Affiliations:** 1 Fiscalia General del Estado, Campeche, Mexico; 2 Universidad Autónoma Campeche, Centro de Investigaciones Biomédicas, Campeche, Mexico; 3 Centro Estatal de Oncología, Campeche, Mexico; 4 Universidad Autónoma Campeche, Facultad de Odontología, Campeche, Mexico

**Keywords:** Trypanosoma cruzi, Metacyclic trypomastigotes, Triatoma dimidiata, Feces, Th subpopulation, Skin, Heart

## Abstract

*Trypanosoma cruzi* infection involves transmission of metacyclic trypomastigotes through injured skin or mucosa via contaminated feces from insect vectors like *Triatoma dimidiata* (Latreille, 1811). Currently, there is insufficient information describing the immune response to feces naturally contaminated with metacyclic trypomastigotes. Mice subcutaneously inoculated with tissue-culture derived trypomastigotes (TCT) or *T. dimidiata* feces containing metacyclic trypomastigotes (MT) or previously multi-exposed (ME) with feces without metacyclic trypomastigotes and then infected with feces containing metacyclic parasites or only *T. dimidiata* feces (F) was studied from 15 min to three months post-infection. PCR detection of parasite DNA at the inoculation site demonstrated persistence of *T. cruzi* DNA up to 20 days in MT and TCT but disappeared earlier in the ME test group. A rapid spread of *T. cruzi* DNA to regional lymph nodes was observed in all experimental groups. A lower amount of amastigote nests in the heart with concomitant intense inflammation was noticed in ME mice in comparison to the MT group. CD4 + T cell subtypes at popliteal lymph nodes shows early Th1 and Th17 responses at seven days in ME mice, whereas Th1, Th17 and Treg predominate in MT mice after three weeks, and feces induces Th1, Th17 and Treg at a later stage. Our study shows that previous exposure to feces prior to infection with *T. cruzi* helps control parasitism at the inoculation site and in heart tissue, and an early induction of Th1 and Th17 T cell subtypes.

## INTRODUCTION

The main route of *Trypanosoma cruzi* infection transmission in endemic areas such as Mexico’s Yucatan peninsula is via *Triatoma dimidiata* feces (Latreille, 1811) contaminated with metacyclic trypomastigotes that penetrate injured skin or mucous membranes^1,2^. It is currently accepted that metacyclic trypomastigotes cannot penetrate intact skin but can access the body via feeding punctures or small, exposed wounds on the skin^3-5^.

Although cyclic transmission of *T. cruzi* is well established, there is insufficient information describing the immune response to feces naturally contaminated with metacyclic trypomastigotes at the inoculation site. It is well understood that vector transmission infection involves metacyclic trypomastigotes present in feces that also contain bacterial microbiota. A recent review highlighted that the inoculum size, the route of infection, *T. cruzi* infective stages, and the relationship between *T. cruzi* and feces microbiota may all be influential factors in infection^6^. In short, successful *T. cruzi* infection depends on many factors, such as: vector species, percentage of naturally infected triatomine (i.e., parasite carriers), number of times of human exposure to triatomine bite, route of infection, inoculum size but, more importantly, triatomine feces contamination with *T. cruzi*.

Immune events occurring immediately after bodily parasite entrance are poorly understood. There is controversy regarding how infection is initiated. In the 1980’s, it was suggested that infection through the triatomine feeding wound was very inefficient^7^, but others have found this route suitable to actively infect^4^, and recently it was demonstrated that low parasitic inoculum is effective in causing infection when metacyclic trypomastigotes gain access through the triatomine feeding wound^5^. Metacyclic trypomastigotes represent the natural infectious phase in vector transmission. However, experimental models in general did not use this phase, but instead used trypomastigotes-tissue-culture derived, or metacyclic trypomastigotes-culture-induced or purified metacyclic trypomastigotes of bacteria-free triatomine feces.

Poncini *et al*.^8^ reported the characterization of the inflammatory response at the parasite inoculation site using 500 (blood-purified) trypomastigotes in murine models. They observed low occurrence of skin infiltration at three or seven days post-infection and no detection of amastigote nests, suggesting *T. cruzi* can silently burrow through mouse skin. Recently, Guitierrez *et al*.^9^ reported that metacyclic trypomastigotes obtained in vitro from epimastigotes and inoculated intradermally are less virulent than blood-derived trypomastigotes; interestingly, amastigote nests associated with skin infection were only detected in mice infected with blood-derived trypomastigotes. However, both studies did not use the natural infectious phase, nor feces in which parasites are naturally found.

The route of experimental inoculation is usually intraperitoneal, but natural infections occur through bite puncture, mucosa, or intradermal exposure. However, there are few studies that meet the above criteria. A recent study demonstrated that mono- or multi-exposure to *T. dimidiata* saliva or feces before inoculation with metacyclic trypomastigotes helped to control the level of parasitemia in *T. cruzi*-infected mice^10,11^.

In this context, it is important to fill this gap in knowledge using natural metacyclic trypomastigotes and triatomine feces in *Trypanosoma cruzi* infection and describing early immune responses at the inoculation site and nearby tissues, including heart muscle tissue. Our main goal is to describe the ongoing inflammatory reaction at the inoculation site and the heart.

## MATERIALS AND METHODS

### Triatoma feces collection and mice inoculation

*Triatoma dimidiata* adult insects were captured in the periphery of Campeche city, Mexico (latitude 19.84386 and longitude −90.52554). *T. dimidiata* species were used because they are the most relevant in the studied location^12-14^. Once in the laboratory, they were fed on mice. After feeding, insect feces and urine were collected and tested for the presence of *Trypanosoma cruzi* flagellates. Negative samples were re-checked by polymerase chain reaction (PCR) with specific primers as reported elsewhere^15,16^. Triatomines were divided into *T. cruzi* positive and negative groups that were then used as the source of feces. In short, feces of one positive triatomine were used to infect mice. Approximately 25 to 30 days post-infection, when parasitemia was confirmed to be high, *T. cruzi*-negative captured triatomines (approximately 15) were fed on the infected mice. These newly infected triatomines were used as the source of contaminated feces. In total, approximately 15 infected (*T. cruzi*-positive) and 15 non-infected (*T. cruzi*-negative) triatomines were available for experimental purposes. Experimental feces were obtained from *Triatoma* after feeding on mice. Approximately 200–600 µL of total feces/urine mixture was collected .The inoculum was adjusted to 1,000 metacyclic trypomastigotes/10 µL in phosphate‐buffered saline (PBS) for experimental purposes.

A total of 160, 8-week-old female BALB/c mice weighing 25.5 g ± 2 g were used in the experiment, separated into four groups. Each experimental group was allocated in separate racks with 10 correctly labeled mice per cage. The control mice group (five animals for each experimental point) were exposed to 10–20 µL of parasite-free feces through subcutaneous inoculation. The sample size of five animals allows for statistical analysis. In all cases, insulin syringes were used to inoculate mice at their hind pads. The metacyclic-mono-exposure group (or “MT group”) was inoculated with 10–20 µL of *Triatoma* feces that naturally contaminated *T. cruzi* metacyclic trypomastigotes (five animals for each experimental point). The inoculum composition per mouse consisted of approximately 1,000 parasites, of which >90% were metacyclic trypomastigotes. Mice were sacrificed 15 min, 1 h, 4 h, 24 h, seven days, 20 days, one month, two months, and three months after inoculation. The multi-exposure group (“ME” group) consisted of animals (five animals for each experimental point) that received 10–20 µL of *Triatoma* feces (free of metacyclic trypomastigotes) inoculum monthly for three months (i.e., a total of three inoculations per mouse). At the end of the 3-month period, the mice were 20 weeks old and weighted approximately 37 g. They were infected with 10–20 µL of *Triatoma* feces with approximatley 1,000 naturally metacyclic trypomastigotes. The mice were then sacrificed after 15 min, 1 h, 4 h, 24 h, seven days, 20 days, two months, and three months. Another study group, called tissue-culture *T. cruzi* (TCT), received 1,000 trypomastigotes obtained from HeLa cell culture infected with *T. cruzi*. Parasites were diluted in Minimum Essential Medium (MEM medium) (Sigma-Aldrich) without fetal calf serum. After inoculation with *T. cruzi* , mice were sacrificed under deep anesthesia ( Rompun^®^, Bayer, Leverkusen, Germany), after 15 min, 1 h, 4 h, 24 h, seven days, 20 days, two months and three months. Time intervals were selected to cover early, acute and chronic inflammatory responses. In all cases, skin from inoculation sites, lymph nodes near inoculation sites (popliteal lymph node) and heart tissues were obtained and immediately used, fixed, or frozen depending on the type of procedure.

### Ethics

Animals were maintained in accordance with the Guide for the Care and Use of Laboratory Animals and the Ethical Principles for Animal Experimentation established by the NORMA Oficial Mexicana NOM-062-ZOO-1999 and the ARRIVE guidelines, after being approved by DGPI project UAC-002-2021.

A total of 160, 8-week old female BALB/c mice weighing 25.5 g ± 2g were used in the experiment and separated into four groups. The animals were housed in plastic cages with white wood chips as bedding in the animal facility of Universidad Autonoma de Campeche (UACAM). They were provided with unrestricted access to a complete commercial meal mixture and tap water. Environment lighting and temperature were strictly regulated, maintaining a temperature of 25 ± 2 °C

### PCR for T. cruzi detection

DNA extraction was carried out using the Wizard Genomic DNA Purification Kit (Promega) following the manufacturer’s instructions. Briefly, a sample of each tissue was homogenized in tissue-culture plates in cell lysis solution, followed by nucleic lysis solution, then RNA degradation and protein precipitation. Thereafter, supernatant was recovered, and DNA was precipitated with isopropanol. The DNA pellet was washed and kept at −20 °C until further use.

Regarding skin samples, a half piece from each animal, which corresponded to its respective experimental time point, was taken and pooled, while the other half was used for histology. The entire lymph node from the five animals corresponding to respective experimental time points were taken and pooled. Heart tissue samples were individually processed.

The PCR reaction was performed in 50 µL containing 1 µL of sample DNA, a set of primers 121 (5-AAA TAATGTACGGGTGAGATGCATGA-3) and 122 (5-GGTT CGATTGGGGTTGGTGTAATATA-3), 0.2 mM of each deoxynucleotide triphosphates (dNTPs), and 2.5 U of Taq polymerase (Invitrogen), 10 mM Tris-HCl, pH 8.8, 50 mM KCl, and 1.5 mM MgCl. The reaction was subjected to 35 PCR cycles: 94 °C for 1 min, and the annealing temperature was 56 °C^15,16^. The amplified product was analyzed to confirm the putative 330 bp product via gel electrophoresis and ethidium bromide staining under UV exposure. Positive and negative controls were included for all gels.

### Cytometry

A pool of popliteal lymph node or regional lymph node near the inoculation site from each experimental group of mice corresponding to specific time points was obtained (seven days, 20 days, and two months). Freshly removed lymph nodes were put over a mesh nylon screen in the presence of RPMI medium supplemented with 1% fetal calf serum and using circular motion and pressing against the mesh nylon with a 3 ml syringe plunger until mostly fibrous tissue remains. The flow through was collected in each well in a final 0.5 ml volume and left for 5 min to enable big fragments sedimentation. Cell suspension was centrifuged and resuspended in 1 ml of RPMI medium supplemented with fetal calf serum then cell viability was determined using trypan blue and counted using a Neubauer chamber and adjusted to 1×10^6^ cells/100 µL.

Four assay tubes with 100 µL each per experimental time point were used. Cells were resuspended in RPMI medium supplemented with fetal calf serum and 0.01% sodium azide. Then, they were incubated for 30 min in the presence of 1 µL/100 µL Befeldrin A cell suspension (100X solution Biolegend) in the dark. Upon harvest, cells were surface stained with the antibodies used for the staining and detection of cell surface and intracellular markers. Overall, 3 µL of monoclonal antibodies is used to stain 1 million cells in 100 µL volume in case of surface markers, but for intracellular markers 5 µL of monoclonal antibodies was used. For surface CD3+ and CD4+ markers, four assay tubes were stained with monoclonal Brilliant violet 421 anti-mouse CD3+ (clone KT3.1.IgG2a, Biolegend) and FITC anti-mouse CD4 (clone 129-9 Rat IgG2a, Biolegend) and incubated for 30 min in the dark. After the washing step with sterile PBS with 1% Stain Buffer (FBS) and 0.01% sodium azide the cells were fixed with 4% of paraformaldehyde-PBS for 15 min in the dark with gentle agitation. For intracellular markers, cell suspensions were permeabilized with 100µL of PBS-Saponin 0.1% for 15 min in the dark then intracellular staining was carried out. For Th cell subpopulations, different assay tubes were used. Brillant violet 510 anti-mouse INF-γ (clone XMG1.2 rat IgG1, Biolegend) for Th1, and for Th17 Brilliant violet 510 anti-mouse IL-17 (clone TC11-18H10.1 rat IgG1, Biolegend), and for Treg Brilliant violet 510 anti-mouse FoxP3 (clone MF-14 rat IgG2b, Biolegend) and Brilliant violet 605 anti-mouse CD25 (clone PC61 rat IgG1, Biolegend). Then cells were incubated for a further 40 min in the refrigerator. Cells were then washed thrice with sterile PBS with 1% FBS and 0.01% sodium azide, and samples were analyzed by the Attune Focusing Cytometer.

**Figure 1:**
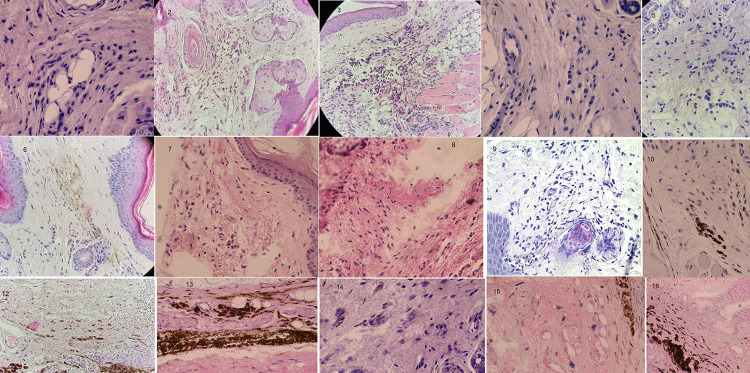
Representative images of histology at inoculation site. Mice were inoculated at hind pad subcutaneously with *Triatoma dimidiata* feces (F) or feces contaminated with metacyclic trypomastigotes (MT) or multi-exposed to parasite-free feces and infected with feces contaminated with metacyclic trypomastigotes (ME) or trypomastigotes tissue culture derived (TCT). The upper panel corresponds to mice inoculated with trypomastigotes tissue culture derived (TCT); the middle panel, to mice inoculated with feces contaminated with metacyclic trypomastigotes; the bottom panel, to mice multi-exposed to parasite-free feces and infected with feces contaminated with metacyclic trypomastigotes. From left to right, it corresponds to 15 min, 1h, 24 h, 5 days and 20 days after inoculation time.

After 30 min incubation in the dark, cell suspensions were washed and analyzed in Attune Focusing Cytometer using channel VL1 for Brilliant violet 421, channel VL2 for Brillant violet 510, channel VL3 for Brilliant violet 605, and channel BL2 for FITC. Before acquisition, an optimization protocol was carried out. Afterwards, unstained and dyed fluorescent beads were used of calibration. Acquisition of 10^4^events were carried out following manufacturer’s instructions. Figure 1 shoes the gating strategy for flow analysis of CD4+/Th subpopulations by flowcytometry; first FSC-H vs FSC-A gated singlet cells, after FSC-A vs SSC-A gated mononuclear population, next VLA1-A (CD3+) vs Count events. This region was used for all possible markers combinations. The percentage of Th subpopulation was expressed as a relative count of total CD4+ cells; each experimental point time was carried out in triplicate. Finally, data were statistically analyzed. One way analysis of variance (ANOVA) Tukey’s multiple comparison test was used to compare data (p<0.05 significance)).

### Histology

The standard procedure of paraffin inclusion and hematoxylin and eosin (H&E) staining was followed. Briefly, after euthanasia of experimental mice, a skin sample at the inoculation site, skeletal muscle, and cardiac tissue were excised and fixed in 10 % buffered formaldehyde-phosphate. Tissue samples were dehydrated and paraffin embedded. The samples were then cut and stained with H&E and observed for autofluorescence using an epifluorescence microscope^17^. Tissue samples were obtained and coded in our laboratory and sent to the Pathology department.

Inflammatory response investigation at the inoculation site was assessed by pathologists based on their morphology and staining pattern in hematoxylin and eosin. In short, after H&E stain, sections were quantified for mast cells (MC) and fibroblast activation. MC were examined for morphological changes such as degranulation and/or metachromatic cytoplasm. Activated fibroblasts have large nuclei size and heterochromatin. Cells were counted in 10 fields each, in areas with evidence of inoculated feces in the dermis under high-power magnification (400x). Results were expressed as the ratio between number of cells/field in five different mice.

Inflammatory responses and amastigote numbers in cardiac tissue were examined in five different mice. Inflammatory focus was considered if at least five mononuclear cells were grouped. Foci and nest amastigotes were counted in 10 fields each at 400X magnification. Results were expressed as the ratio of foci or nests/field at 400x magnification.

### Statistical analysis

Descriptive statistics frequencies and percentages were used to summarize data. Kruskal-Wallis test was used for multiple comparisons for numerical variable differences between groups and were expressed as mean ± standard error. One way ANOVA Tukey’s multiple comparison test was used to compare data (p<0.05 significance). The program used was GraphPad Prism (version 10.2, GraphPad Software, Boston, MA, USA).

## RESULTS

### Parasite persistence at the inoculation site and tissue dissemination

*T. cruzi* DNA from 15 min to 20 days after initial inoculation was positive in all experimental groups, except for the multi-exposure (ME) group, which was negative for the presence of *T. cruzi* DNA up to 20 days. However, one and three months post-inoculation, *T. cruzi* DNA was not detected at the inoculation site in any experimental group (Table 1).

**Table 1:** PCR demonstration of parasite persistence at the inoculation site and tissue dissemination.

	Trypomastigotes-culture derived (TCT)	Metacyclic trypomastigotes-feces (MT)	Multi-exposed to feces and infected with trypomastigotes-feces (ME)
Skin 15 min	Positive	Positive	Positive
Skin 1 h	Positive	Positive	Positive
Skin 4 h	Positive	Positive	Positive
Skin 24 h	Positive	Positive	Positive
Skin 7 days	Positive	Positive	Positive
Skin 20 days	Positive	Positive	Negative
Skin 1 month	Negative	Negative	Negative
Skin 2 months	Negative	Negative	Negative
Skin 3 months	Negative	Negative	Negative
Heart 15 min	Negative	Negative	Negative
Heart 1 h	Negative	Negative	Negative
Heart 24 h	Negative	Negative	Negative
Heart 7 days	Negative	Negative	Positive
Heart 1 month	Positive	Positive	Positive
Heart 2 months	Positive	Positive	Negative
Heart 3 months	Positive	Positive	Negative
Lymph node 15 min	Positive	Positive	Positive
Lymph node 1 h	Positive	Positive	Positive
Lymph node 24 h	Positive	Positive	Positive
Lymph node 7 days	Negative	Positive	Positive
Lymph node 20 days	Positive	Positive	Positive
Lymph node 1 month	Negative	Negative	Positive
Lymph node 2 months	Negative	Negative	Positive
Lymph node 3 months	Negative	Negative	Positive

*T. cruzi* DNA dissemination to the heart was evident as early as seven days post-inoculation in the ME group, but negative in other experimental groups. Notably, no evidence of inflammation was detected at this experimental time point (seven days post-infection) in any experimental group (data not shown). At one month post-inoculation, all experimental mice, independent of the parasite’s pha7se, were positive for *T. cruzi* DNA and showed clear evidence of nesting of amastigotes with inflammatory response. After three months of infection, the presence of parasite DNA could only be detected in the MT mice whereas in TCT and ME groups were negative (Table 1).

Parasite DNA dissemination to regional lymph nodes was noted as early as 15 min after inoculation and throughout 20 days post-inoculation in all experimental mice; only the ME group remained *T. cruzi* DNA positive after the acute phase of infection two and three months post-inoculation (Table 1).

### Inflammatory response at the inoculation site and cardiac tissues

We focused on fibroblast and mast cell activation modalities in investigating the inflammatory response at the inoculation site. A sign of mast cell activation was observed as early as 15 min and throughout 1 h after inoculation of parasite-free or muti-exposed (MT, ME) feces compared to the TCT group. After 24 h of inoculation, mast cell activation was higher in TCT mice compared to ME and MT groups, but similar activation between ME and MT groups. After seven and 20 days of inoculation, mast cell activation was higher in mice inoculated with parasite-free feces compared to ME, MT and TCT groups (Figure 1). Regarding fibroblast activation, we noticed a rapid reaction as early as 15 min after inoculation, especially in mice inoculated with feces contaminated with metacyclic trypomastigotes, but not in TCT-inoculated mice. However,1 h after inoculation, fibroblast of mice in the TCT group were activated in a similar fashion to that of the other groups. However, five days post-inoculation, the TCT group manifested decreased fibroblast activation, whereas the rest of experimental groups sustained activation and collagen disruption (Figure 1).

**Figure 2:**
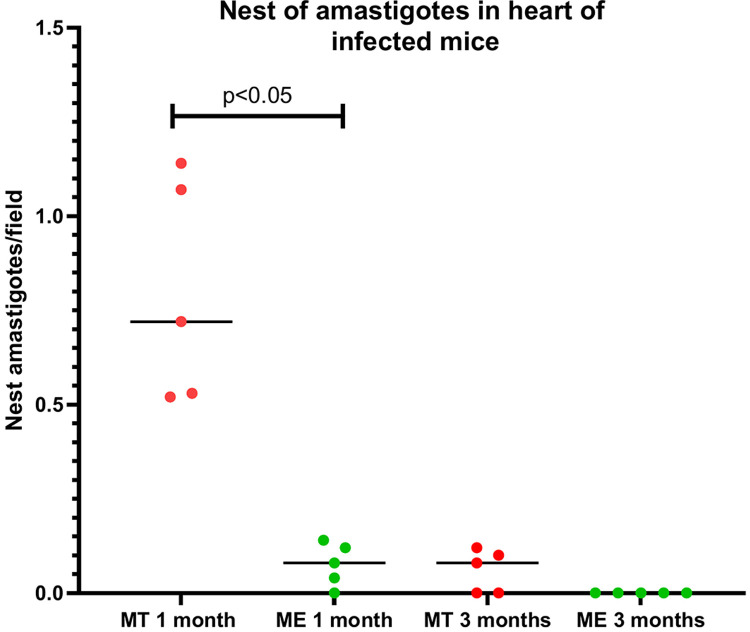
Graph of number of amastigote nests in heart tissue. Number of nests of amastigotes/field in heart at one and three months post-infection. At least 10 fields per mouse and five mice per experimental time were analyzed. Kruskal-Wallis test multiple comparison was used to compare data (p<0.05 significance).

**Figure 3:**
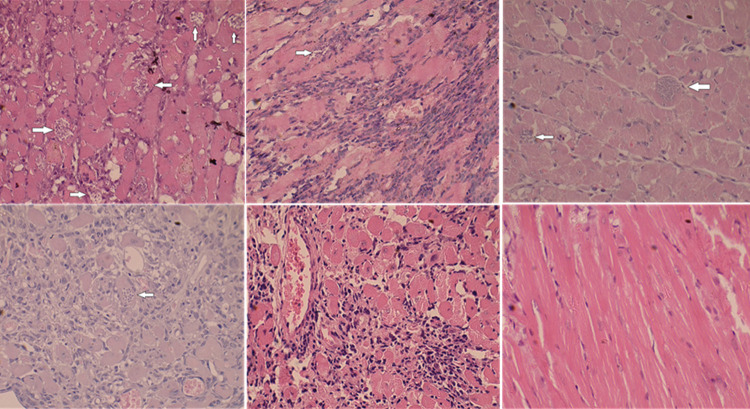
Representative images of heart histology. Mice were inoculated at hind pad subcutaneously with *Triatoma dimidiata* feces (F) or feces contaminated with metacyclic trypomastigotes (MT) or multi-exposed to parasite-free feces and infected with feces contaminated with metacyclic trypomastigotes (ME) or trypomastigotes tissue culture derived (TCT). After one month, and three months post inoculation, heart tissue was processed for hematoxylin-eosin stain and observed at 400X. Arrows indicate the presence of amastigote nests. From left to right, it corresponds to the MT, ME and TCT groups; the upper panel corresponds to one month post-infection and the bottom panel to three moths post-infection

The inflammatory reaction in the heart tissue seems to be dependent on parasite’s phase. That is, less myositis and myocytolysis was induced by trypomastigotes-tissue cultured derived parasites, whereas the use of metacyclic trypomastigotes induced stronger myositis and myocytolylis. The number of amastigote nests one month post-infection was lower and statistically different between mice that were first exposed to feces for a prolonged period and then infected with metacyclic trypomastigotes (0.1 nest/field), versus mice inoculated with feces containing metacyclic trypomastigotes (0.7 nest/field). Similarly, a lower number of nests was observed in mice inoculated with trypomastigote tissue-culture derived parasite (0.05 nest/field). Three months after *T. cruzi* infection, the number of amastigote nests fell to 0.1 nests/field in MT whereas in ME amastigote nests were not detected (Figure 2); myocarditis was notably lower in mice inoculated with TCT whereas myocarditis remained evident in mice inoculated with Triatoma feces contaminated with or without metacyclic trypomastigotes. (Figure 3).

### Th cells subpopulations induced at regional lymph nodes

**Figure 4:**
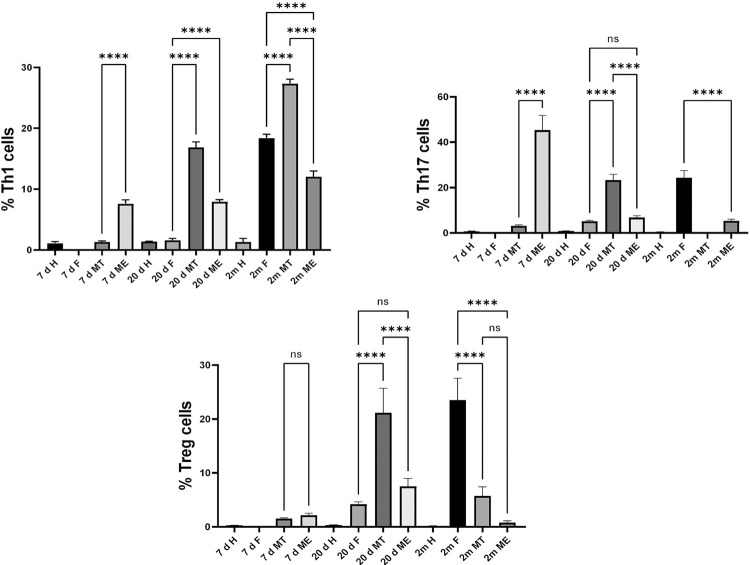
CD4+/T helper subpopulation at the popliteal regional lymph node after intradermal *Trypanosoma cruzi* inoculation. Mice were inoculated at hind pad with *Triatoma dimidiata* feces (F); or feces contaminated with metacyclic trypomastigotes (MT), or multi-exposed to parasite-free feces and infected with feces contaminated with metacyclic trypomastigotes (ME), Healthy (H). Lymph node CD4+ analyzed after seven days, 20 days, and two months post-inoculation.; Anova test multiple comparison was used to compare data among groups (**** p<0.0005 ).

Supplementary Figure S1 shows the gating strategy for the flow analysis of lymph node CD4+/Th subpopulations by flowcytometry. Th1 and Th17 subpopulations increased in mice previously inoculated with parasite-free feces and then infected with feces contaminated with metacyclic trypomastigotes at one week after initial inoculation. Three weeks post-inoculation, the highest activation of Th1 and Treg subpopulations occurred in the group of mice mono-exposed to feces contaminated with metacyclic trypomastigotes (MT) whereas in the ME mice the Th1 response was sustained. Two months post-inoculation, activation of Th1 and Treg subpopulations was evident in mice inoculated with feces. (Figure 4). Feces induce activation of Th1 and Th17 as early as three weeks post-inoculation and remained active during two months with a marked increase in Treg cell types at the two-month mark. Overall, the CD4+ Th1 subpopulation was more prone to activation in comparison to Th17 and Treg subpopulations.

## DISCUSSION

The infection with *T. cruzi* via vector transmission involves triatomine feces that contain bacterial microbiota and metacyclic trypomastigotes. In nature, successful *T. cruzi* infection depends on many factors, such as: vector species, naturally infected triatomine percentage (i.e., carriers of the parasite), number of times of human exposure to triatomine feces, route of infection, inoculum size but more importantly, triatomine feces contamination with *T. cruzi* ^1-6^. However, there are few reported studies on the inflammatory response to triatomine feces that contain bacterial microbiota and metacyclic trypomastigotes. In this study, we analyzed the inflammatory response at the inoculation site and nearby tissues proximal to inoculation and re-inoculation sites of *T. dimidiata* feces naturally infected with *T. cruzi* or trypomastigotes inoculum derived from cell culture and, lastly, parasite dissemination from the inoculation site. Recently, it has been published the highly abundant and diverse genus and species of *T. dimidiata* gut bacteria recognizing Corynebacterium, Tsukamurella, Brevibacterium, and Staphylococcus as the most abundant microorganisms. Also, these papers describe the interaction between microbiota, the insect itself, including how *T. dimidiata* microbiota may play an active role in modulating parasite development^18-20^.

The events occurring immediately after *T. cruzi* deposition on injured skin remain poorly investigated and understood. We observed that parasite DNA can persist at the infection site for up to three weeks after inoculation if the inoculum used was *T. cruzi*-tissue culture derived (TCT) or from triatomine feces contaminated with metacyclic trypomastigotes (MT). However, in animals previously inoculated with *T. dimidiata* feces that were free of *T. cruzi* then later infected with MT-contaminated *T. dimidiata* feces, *T. cruzi* DNA was undetectable during this timeframe. Three weeks post-inoculation, DNA was no longer detected in TCT and MT experimental groups. These data suggest that *T. cruzi* and/or DNA parasite disappear shortly after initial inoculation. However, if animals were exposed to triatomine feces before infection, DNA clearance happened sooner. In a recent study, it was reported that very low levels of DNA at the inoculation site can persist for up to 12 days if metacyclic trypomastigotes obtained by one round of differentiation from epimastigotes were used^8,9^. These data suggest that the parasite did not persist for long periods of time at the inoculation site regardless of the *T. cruzi* strain or infective metacyclic stage used. Unfortunately, a limitation in our study is that we did not quantify parasite DNA between experimental groups.

In our work, histological skin samples analyses at the inoculation site did not reveal the presence of nest amastigotes at any experimental time point. However, an intense inflammatory reaction comprised of mononuclear cells happened 15 min after inoculation in mice that were previously pre-exposed with triatomine *T. cruzi*-free feces and then infected with MT-triatomine feces. However, other studies have reported the presence of nest amastigotes and relatively lower inflammatory reactions even 30 days after infection with blood stream trypomastigotes^8,9^. This discordant inflammatory reaction may be explained because in our research we used triatomine feces rich in bacteria that are able to display strong immune cell recruitment to the site of inoculation^10,21,22^. Nevertheless, we do not have a clear explanation for the presence of nest amastigotes at the inoculation site. But previous researchers have suggested that *T. cruzi* triggers recruitment of M_o_-derived cells with dual action mechanism during the course of the infection to not only control the parasite from spreading but also to minimize its persistence^8^.

In multi-exposed mice to triatomine feces before infection, parasite DNA cleared more quickly than in other groups. It is possible that the macrophages-high inflammatory reaction, along with Th17 subpopulation profile peaking at seven days after infection, may contribute to the elimination and reduction of parasitic inoculum load, and consequently limit the persistence of parasites at the inoculation site and progress of the infection. Unfortunately, we did not measure the number of parasites to support our hypothesis. However, a recent investigation reported that lipopolysaccharides (LPS) could activate dendritic cells, promoting CD8+ T cell response and therefore reduce parasitemia conferring partial protection against infection with *T. cruzi* ^21^. Similar results were recently observed in bacterial LPSstimulation in the dermis prior to *Trypanosoma brucei* sp. infection in a murine model, they found LPStreatment enhances the ability of dermal macrophages to eliminate intradermal injected *T. brucei* parasites in the skin^22^. Additionally, studies on *T. dimidiata* microbiota have demonstrated the presence of Gram-positive and Gram-bacteria rich in LPS^18,19^.

While authoring our study results, an article was published where they analyzed early events at the inoculation site after *T. cruzi* intradermal inoculation. They found that CD45+ cells were the predominant cell types at the inoculation site and parasites actively proliferated during the first 10 days post-infection. Decline in parasite load was dependent on the ability of the host to produce gamma interferon, but delayed activation of T cells facilitated *T. cruzi* establishment^23,24^. Our findings showed Th1 and Th17 subpopulations as early as one week and until day 20 after infection. Their data align with and complement our finding, despite their use of higher trypomastigotes-tissue culture derived parasite inoculum instead of using metacyclic stage. In both studies, parasites disappeared quickly from the inoculation site (i.e., 10 days in our study and three weeks in theirs), but pre-exposure with triatomine feces induced an intense mononuclear inflammatory reaction as early as 1-hr post-inoculation with a Th17 profile. Thus, immune responses to feces microbiota may have a bystander effect on metacyclic trypomastigotes reducing parasite infectivity.

Conversely, the presence of edema, collagen fragmentation and mast cell activation as early as 1 h post-inoculation at the infection site in MT and ME may assist parasites in penetrating deeper into the dermis and consequently disseminating to other tissues through blood or lymph vessels. In previous work, it has been reported that *T. cruzi* is able to induce edematogenic responses and *in vitro* parasites can transmigrate across endothelial cells^25^. The presence of parasite DNA in regional lymph nodes proximal to the inoculation site was detected as early as 20 min post-inoculation and clearly shows rapid spread. This rapid dissemination may have several possibilities such as phagocytosis of parasites (via Langerhans cells) or dendritic cells, which are sentinels patrolling and transporting antigens to the lymph nodes^26,27^. Alternatively, parasites may migrate through the dermis until reaching lymph node vessels and transmigrating via endothelial cell junctions^28^. Lastly, liquid inoculum can produce sufficiently high hydrostatic pressure that may favor parasite dissemination directly through and into lymph vessels.

Dissemination or replication of *T. cruzi* in heart tissue was observed as soon as seven days post-inoculation in the group of mice (i.e., ME experimental group) previously inoculated with *T. dimidiata* feces free of *T. cruzi* and infected with MT-Triatoma feces, but not in other groups. The possible explanation for the presence of parasite DNA in the heart seven days after inoculation may be the parasite’s ability to escape more rapidly from a dermis with profound tissue remodeling such as collagen fragmentation and edema as was observed in the infected mice population; similar data was observed by Padilla *et al*.^24^. However, parasites persisted in the heart tissue up to three months (last experimental time point in this investigation) after initial inoculation with MT-triatomine feces, but not in ME, in which parasites disappeared sooner. The ME group presented lower amastigote nests than the MT group but both had higher myocarditis and heart damage in contrast to the TCT group. The aforementioned observations may suggest that infection with metacyclic trypomastigote induces a more intense inflammatory reaction and parasite replication than the one observed in mice infected with trypomastigotes derived from cell culture. It has been reported that different stages of the same strain of *T. cruzi* are differentially recognized by the immune system and elicit a different pathology^29^. Furthermore, the same strain and stage of infection but with previous immunization or pre-exposure with triatomine feces, as described in the ME group, may help control tissue parasitism and overall parasite load at the inoculation site, but do not protect from tissue damage.

In our data, we demonstrated that mice inoculated with triatomine feces naturally contaminated with metacyclic trypomastigotes demonstrated dominant Th1 and Th17 profiles, but notably Treg subpopulation persisted for three weeks post-infection and remained at low levels during infection. This profile explains the persistence of inflammation and decreased nests amastigotes in muscle tissue.

Possibly, the specificity of Th1 and Th17 subpopulations in animals previously immunized with triatomine feces and then infected with triatomine feces naturally contaminated with metacyclic trypomastigotes (ME) mice could be directed to feces microbiota antigens. Previous evidence has been reported that triatomine feces can induce local inflammation and specific immune responses^10^. In this context, previous contact with triatomine feces free of metacyclic trypomastigotes and then later infected with feces contaminated with metacyclic trypomastigotes, modified the inflammatory response at the inoculation site. It may decrease the inoculum burden and consequently the severity and parasite persistence in tissues as well. However, this hypothesis needs to be demonstrated in further studies.

In previous work, it has been demonstrated that reinfection with metacyclic trypomastigotes in animal models induced expression of IL-2, IL-4, IL-12, and TGF-beta in regional lymph nodes near the inoculation site. Also, parasite DNA was no longer detectable 24 h after initial infection at the inoculation site while an inflammatory reaction in heart tissue increased in severity 24 h after parasite re-entry, peaking on the 7^th^ day^30,31^ suggesting a possible role of autoimmunity. In our study, we did not explore this mechanism.

This study investigated Chagas disease in endemic areas where *T. dimidiata* is the main vector. Interestingly, we have found low seroprevalence anti-*T cruzi* antibodies and cardiopathy in humans and high frequency of human blood in *T. dimidiata* ^2^. This low seroprevalence is probably associated to humans having persistent contact with *T. dimidiata* feces free of *T. cruzi* contamination. Thus, individuals may develop an immune response to *T. dimidiata* microbiota first and when exposed to *T. dimidiata* feces contaminated with metacyclic trypomastigotes may present a bystander effect on metacyclic trypomastigotes reducing the parasite load and consequently the severity of the disease.

## CONCLUSION

In mice inoculated with *T. dimidiata* feces appears to have induced Th1 and Th17 subpopulations in lymph nodes at and a local inflammatory response at the inoculation site. In mice inoculated with *T. dimidiata* feces naturally contaminated with metacyclic trypomastigotes (MT) the parasite DNA remained detectable at the inoculation site up to 20 days after inoculation with predominantly a Th1 and Th17 subpopulation up to three weeks. However, in mice multiexposed to *T. dimidiata* feces free of parasites and then infected with feces contaminated with metacyclic trypomastigotes (ME), parasite DNA disappeared early and was similarly accompanied with mixed subpopulations of Th1 and Th17 during the first week. Intense myocarditis and myocytolysis was observed in both experimental groups infected with metacyclic trypomastigotes. Furthermore, in mice (i.e. ME group) previously inoculated with *Triatoma dimidiata* feces, less relative parasitism was observed, suggesting that prior exposure to *T. dimidiata* feces alter the course and severity of infection. However, further studies need to be done to quantify parasite load in different tissues and investigate which bacteria are found and how they interact with the host immune system.




