# Supplementary material for: Immunomodulatory effects of Triatoma dimidiata feces on Trypanosoma cruzi infection in a murine model
Source: Rev Inst Med Trop Sao Paulo. 2025 Feb 3;67:e5. doi: 10.1590/S1678-9946202567005 (PMC11790074; doi:10.1590/S1678-9946202567005)
Supplement: Supplementary file 1 [file 1678-9946-rimtsp-67-S1678-9946202567005-suppl01.pdf]

REVISTA DO INSTITUTO DE MEDICINA TROPICAL DE SÃO PAULO

JOURNAL OF THE SÃO PAULO INSTITUTE OF TROPICAL MEDICINE

SUPPLEMENTARY MATERIAL

https://doi.org/10.48331/scielodata.FIMIZF

Immunomodulatory effects of *Triatoma dimidiata* feces on *Trypanosoma cruzi* infection in a murine model

Sergio Escobar-Laines [1], Victor Monteon [2], Carlos Ramírez-Sarmiento [2], Verónica Macedo-Reyes [3], Floribeth León Pérez [4]

[1]Fiscalia General del Estado, Campeche, Mexico

[2]Universidad Autónoma Campeche, Centro de Investigaciones Biomédicas, Campeche, Mexico

[3]Centro Estatal de Oncología, Campeche, Mexico

[4]Universidad Autónoma Campeche, Facultad de Odontología, Campeche, Mexico

Correspondence to: Victor Monteon Universidad Autónoma Campeche, Centro de Investigaciones Biomédicas, Av. Agustín Melgar, S/N entre Calle 20 y Juan de la Barrera, Col. Buenavista, CP 24039, Campeche, Mexico

E-mail: victormonteon@yahoo.com.mx

Received: 8 October 2024

Accepted: 9 December 2024

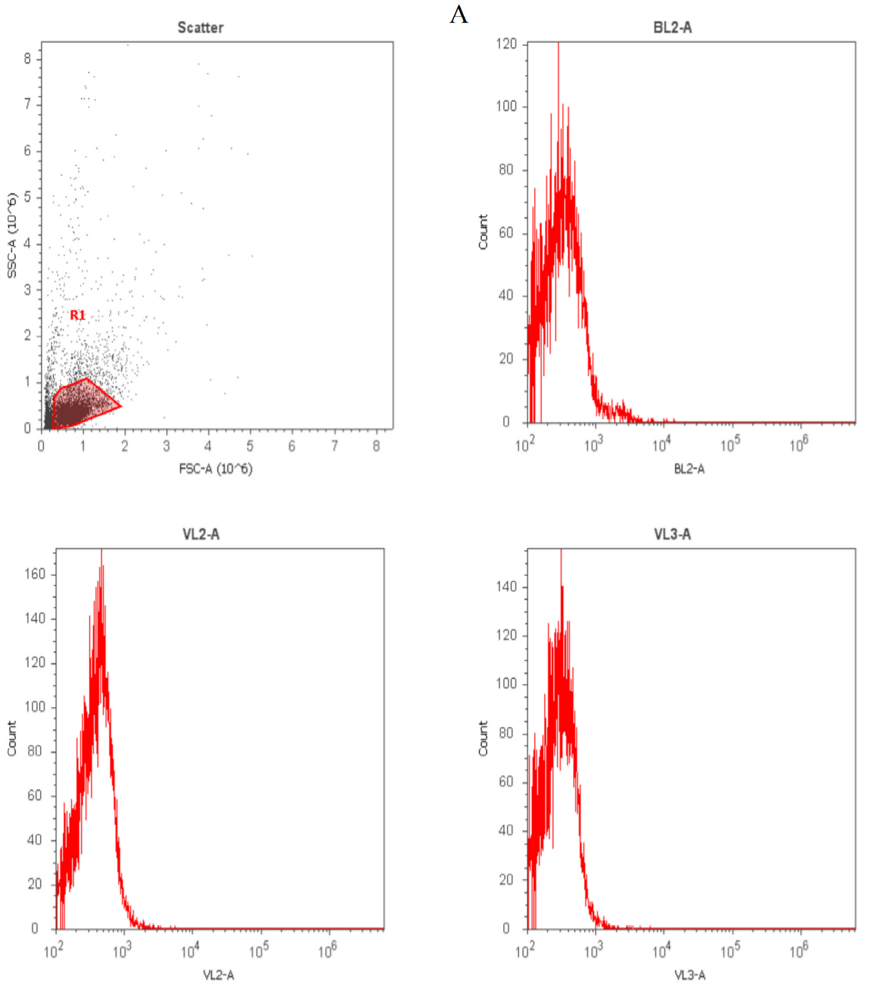

Supplementary Figure S1 - Gating strategy for the flow analysis of lymph node CD4+/Th subpopulations. Gating strategy for the flow analysis of lymph node CD4+/Th subpopulations by flowcytometry. Acquisition and analyses were performed using Attune Acoustic Focusing Cytometer Software: Panel A represents unstained cells to control for background autofluorescence in the three channels used.

This is an open-access article distributed under the terms of the Creative Commons Attribution License.

B

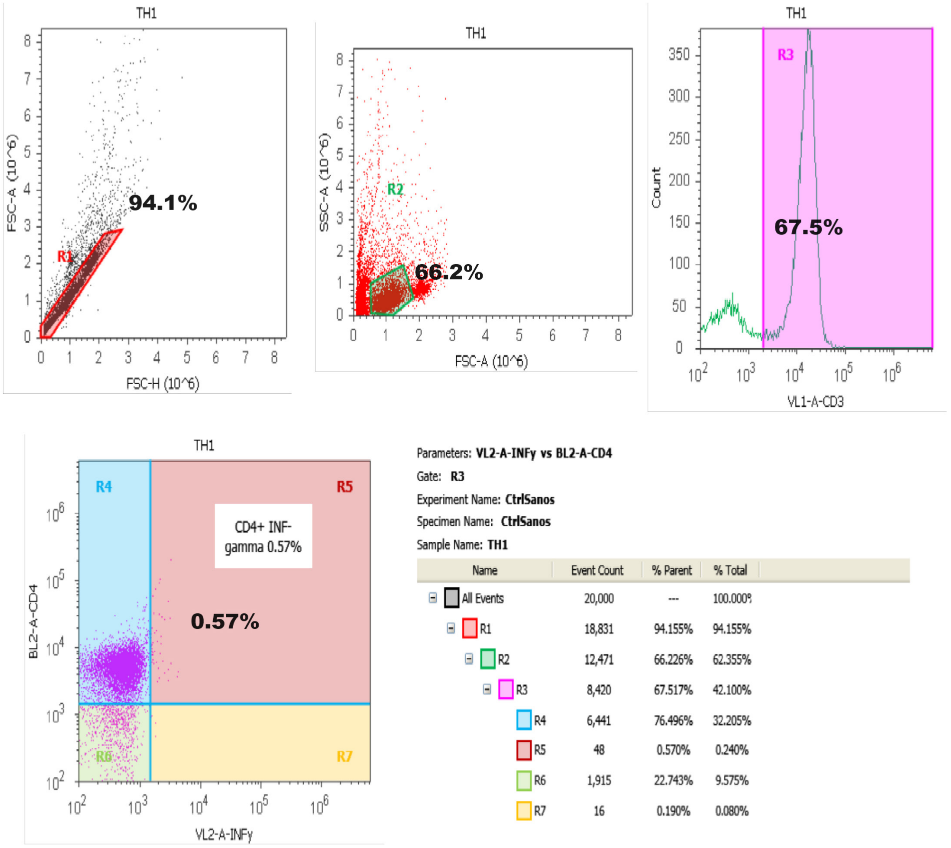

Supplementary Figure S1 - Gating strategy for the flow analysis of lymph node CD4+/Th subpopulations. Gating strategy for the flow analysis of lymph node CD4+/Th subpopulations by flowcytometry. Acquisition and analyses were performed using Attune Acoustic Focusing Cytometer Software: Panel B is a representative image of subpopulation of healthy control CD+/CD4+/IFN-gamma+. Gates and regions (FSC-H vs FSC-A) for singlets, (FSC-A vs SSC-A) for size vs granularity used to identify mononuclear cells, CD3+/counts for region of CD3+ cells and region of CD3+ for CD4+/IFN-gamma+ for Th 1cells subpopulation.
